# Evolution of macular atrophy in eyes with neovascular age-related macular degeneration compared to fellow non-neovascular eyes

**DOI:** 10.1007/s00417-023-06168-0

**Published:** 2023-08-11

**Authors:** Styliani Blazaki, Emmanouil Blavakis, Gregory Chlouverakis, Georgios Bontzos, Irini Chatziralli, Georgios Smoustopoulos, Eleni Dimitriou, Anastasios Stavrakakis, Stamatina Kabanarou, Tina Xirou, Demetrios G. Vavvas, Miltiadis K. Tsilimbaris

**Affiliations:** 1 Department of Ophthalmology, The University of Crete Medical School, 71110 Voutes, Greece; 2 Laboratory of Biostatistics, Faculty of Medicine, University of Crete, Heraklion, Greece; 3 Department of Ophthalmology, Korgialenio-Benakio General Hospital, Athens, Greece; 4 2nd Department of Ophthalmology, National and Kapodistrian University of Athens, Athens, Greece; 5 Massachusetts Eye and Ear, Department of Ophthalmology, Harvard Medical School, Boston, MA USA

**Keywords:** Age-related macular degeneration, Macular atrophy, Macular progression, Macular incidence

## Abstract

**Purpose:**

Τo evaluate the evolution of macular atrophy (MA) in patients with neovascular AMD (nAMD), compared with their fellow eyes exhibiting dry AMD (dAMD).

**Methods:**

This retrospective study included 124 patients from three centers treated with anti-VEGF in their nAMD eye and having dAMD in the fellow eye. Patients without MA at baseline were analyzed to study the time to first MA development. Synchronous and unsynchronous time course of MA was also studied. MA was evaluated using near-infrared images, while all available optical coherence tomography (OCT) images were used to confirm the criteria proposed by the Classification of Atrophy Meetings group for complete MA.

**Results:**

MA first detection in nAMD eyes increased significantly from year 2 to 6 compared to dAMD eyes. Over the study’s follow-up, 45.1% of nAMD-E developed MA, compared to 16.5% of fellow eyes (*p* < 0.001). When MA in the two eyes was compared in a synchronous paired manner over 4 years, nAMD eyes had an average MA progression rate of 0.275 mm/year versus 0.110 mm/year in their fellow dAMD eyes. Multivariate ANOVA revealed significant time (*p* < 0.001), eye (*p* = 0.003), and time-eye interaction (*p* < 0.001) effects. However, when MA did develop in dAMD eyes and was compared in an asynchronous manner to MA of nAMD eyes, it was found to progress faster in dAMD eyes (dAMD: 0.295 mm/year vs. nAMD: 0.176 mm/year) with a significant time-eye interaction (*p* = 0.015).

**Conclusions:**

In this study, a significant difference in MA incidence and progression was documented in eyes with nAMD under treatment, compared to fellow eye exhibiting dAMD. Eyes with nAMD tended to develop more MA compared to fellow dAMD eyes. However, when atrophy did develop in the fellow dAMD eyes, it progressed faster over time compared to MA in nAMD eyes.


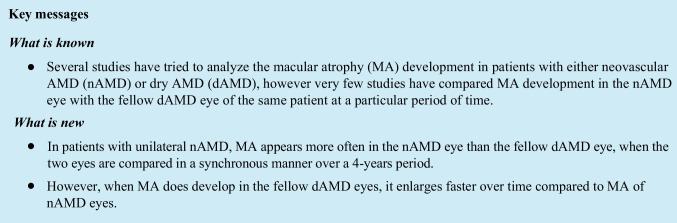


## Introduction

Age-related macular degeneration (AMD) is the leading cause of legal blindness in developed countries and is predicted to affect 288 million people worldwide by 2040 [1–3]. The disease is classified into early, intermediate, and advanced forms, with the latter including choroidal neovascularization (CNV) or central geographic atrophy (GA) [4]. Intravitreal injections of anti-vascular endothelial growth factor (anti-VEGF) agents are considered the gold standard in the treatment of neovascular AMD (nAMD), showing improvement or at least maintenance of visual acuity and anatomical restoration in the majority of patients with nAMD [5, 6].

Despite the proven efficacy of anti-VEGF agents in nAMD treatment, the Comparison of AMD Treatment Trials (CATT) study found that GA was developed in approximately 18% of patients with nAMD treated with anti-VEGF agents for 2 years, and reached 38% at year 5 [7, 8]. Several more recent studies have shown similar results, highlighting atrophy as a major player of visual impairment in eyes with nAMD even when the neovascular component is controlled [9–16].

Thus, the study of atrophy development and evolution has gained self-evident importance both in nAMD and non-nAMD eyes. Several baseline factors have been associated with GA development in eyes with nAMD under anti-VEGF treatment, including the absence of subretinal fluid (SRF), the presence of intraretinal cysts, reticular pseudodrusen, type III CNV, nascent GA, increased central foveal thickness and drusen volume, and the collapse of pigment epithelium detachment, as well as the presence of atrophy in the fellow eye [10–14], while the role of the number and frequency of injections remains controversial [11, 13, 15, 17]. Similarly, the progression of atrophy in dry AMD (dAMD) patients has been associated with such factors as the existence of non-subfoveal (vs subfoveal) MA, the multifocal (vs non-multifocal) pattern of atrophic lesions at baseline, fundus autofluorescence patterns, and hypofluorescent area surrounding by hyperfluorescent margins attributing to lipofuscin concentration and structural abnormalities at the junctional zone of macular atrophy including irregular retinal pigment epithelium elevations and increased inner nuclear layer thickness [18, 19].

In an effort to understand the dynamics of atrophy, some researchers have tried to compare its progression in eyes with nAMD versus eyes with dAMD [18, 20]. Typically, in these studies, the group of nAMD patients is different from this of dAMD patients. However, given the multifactorial nature of AMD, the proper matching of the groups is extremely hard. Until today, very few studies addressed a “within-subjects” approach for the comparison of nAMD and dAMD eyes which would eliminate several confusing factors [21].

In light of the above, the purpose of this study was to evaluate the evolution of macular atrophy in the eyes of patients with nAMD treated with anti-VEGF agents, in comparison to their fellow dAMD eyes.

## Patients and methods

### Data collection

In this retrospective study, patients’ data were collected by three different retinal departments; University Hospital of Heraklion, General Hospital of Athens Korgialenio-Benakio, and Attikon University Hospital of Athens.

Patients with nAMD in one eye and with dAMD in the fellow eye, with at least 2 years of follow-up, were enrolled in the study. In cases that the fellow eye developed nAMD after 2 years of follow-up during the study, the patient’s data were used for the analysis until the time point of neovascularization (CNV) appearance. “Baseline” was defined as the time point when patients were examined for the first time in our departments. Diagnosis of nAMD and dAMD was confirmed using spectral domain optical coherence tomography (SD-OCT) and fundus fluorescein angiography (FFA) or indocyanine green angiography (ICGA) (Spectralis HRA + OCT; Heidelberg Engineering, Inc., Heidelberg, Germany) to evaluate the presence of exudative features in nAMD eyes or clinical features such as drusen, retinal pigment epithelium (RPE) changes, and macular atrophy in dAMD eyes. Patients that had received photodynamic therapy and laser photocoagulation were excluded from the study. In addition, patients with secondary CNV due to other causes, such as pathologic myopia, choroidal rupture, angioid streaks, and ocular histoplasmosis syndrome, or other fundus co-morbidities, such as diabetic retinopathy, retinal vascular occlusions, pattern dystrophy, uveitis, endophthalmitis, and postoperative cystoid macular edema were excluded from the study. Moreover, patients that had undergone any type of posterior segment surgery were not included. The eyes with nAMD were treated with either bevacizumab, ranibizumab (Lucentis; Novartis), aflibercept (Eylea; Bayer), or a combination of those, following a pro re nata (PRN) or a treat and extend (TREX) protocol.

Demographic data, such as age and sex, were extracted from patients’ records. Best-corrected visual acuity (BCVA) measurements were recorded in the Logarithm of the Minimum Angle of Resolution (logMAR) at the first and final visit examination for both eyes of each patient. The total number of intravitreal anti-VEGF injections in each eye during the follow-up period was documented as well as the number of injections that some patients received before the beginning of the study. The duration of follow-up of each patient was measured in years. The CNV lesion was characterized as type I (occult CNV), type II (classic CNV), or type III (retinal angiomatous proliferation) based on FFA and ICGA when available [22].

Near-infrared reflectance (NIR) and SD-OCT images of patients were acquired after pupil dilation in both eyes. Only patients with good-quality images of all time points of interest (baseline and yearly thereafter ± 2 months) were included in the study. In all three centers, imaging was done by experienced and certified persons. SD-OCT scans covered a 6 × 6 mm square centered over the fovea using either a radial or a raster scan pattern for all time points of the study period. Prior to the analysis, interobserver agreement of MA area measurement was performed. Therefore, 20 randomly selected cases of NIR and SD-OCT images of each department (60 images in total) were evaluated by independent graders of each participating center (3 graders) to access interobserver agreement.

### Macular atrophy measurement

**Fig. 1:**
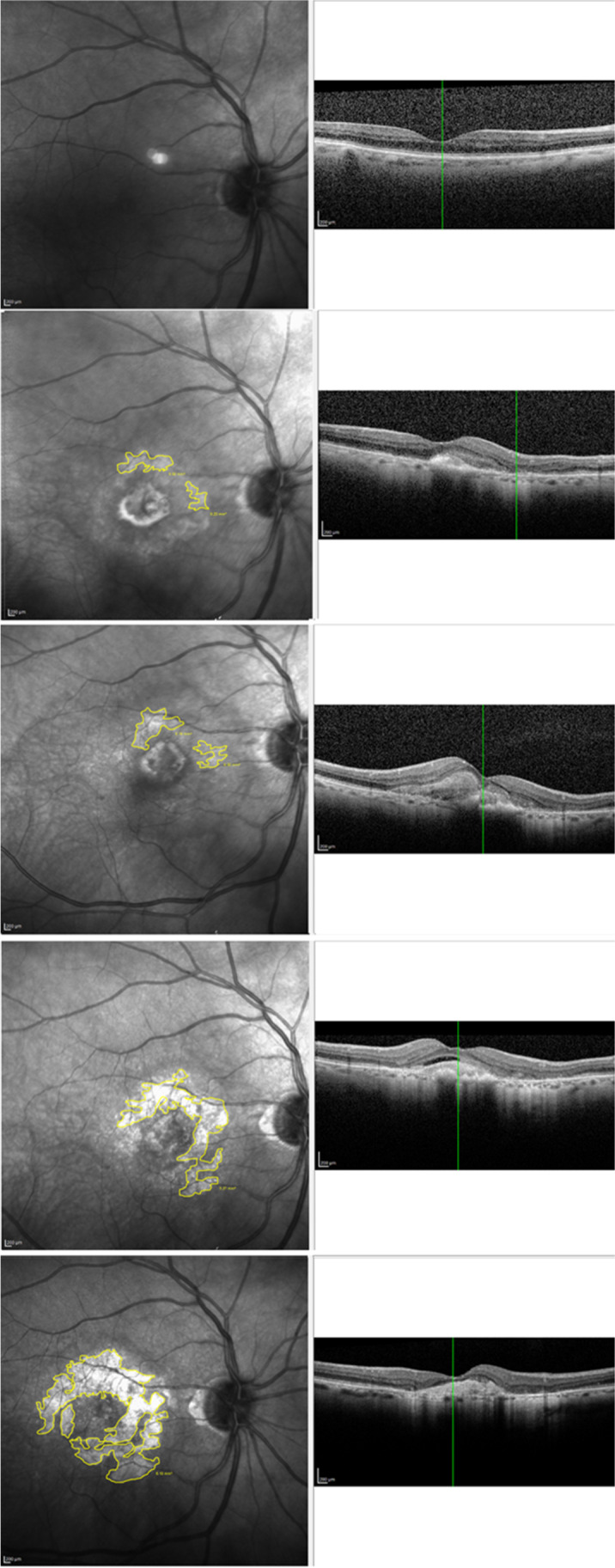
Macular atrophy progression in a patient with neovascular age-related macular degeneration over a period of 4 years. Typical presentation of atrophy in near-infrared (NIR) and optical coherence tomography (OCT)

Macular atrophy was calculated using NIR images, while SD-OCT volume images were used to confirm compliance with CAM group criteria of all available scans in atrophic areas. NIR images were examined first and hyperreflective patches with sharply demarcated edges of a minimum region of 250 μm were located and considered as atrophic [23]; then, graders scrolled through all available OCT B-scans to verify the presence of CAM criteria for complete MA (cRORA) in all scanned hyperreflective areas. These areas were then manually outlined taking into account hypertransmission and OCT scan information. For each NIR image, the sum of all outlined areas was calculated and considered as macular atrophy for the specific time point. CAM group proposed 4 categories for the classification of atrophy associated with AMD on OCT images: complete RPE and outer retinal atrophy (cRORA), incomplete RPE and outer retinal atrophy (iRORA), complete outer retinal atrophy (cORA) and incomplete outer retinal atrophy (iORA). In this study, we used the definition of cRORA to evaluate the scanned areas for atrophy. According to the CAM group, complete MA or cRORA is defined on OCT as an area with (1) choroidal hypertransmission, (2) attenuation or disruption of RPE, and (3) collapse or thinning of outer retinal layers. Figure 1 shows an NIR image with a demarcated atrophic area that fulfilled the criteria of cRORA in all available OCT scans. Cases of RPE loss as a result of RPE tear were excluded. This method using NIR in combination with OCT has been also reported in other studies [11, 13, 23–26].

### Macular atrophy analysis

**Fig. 2:**
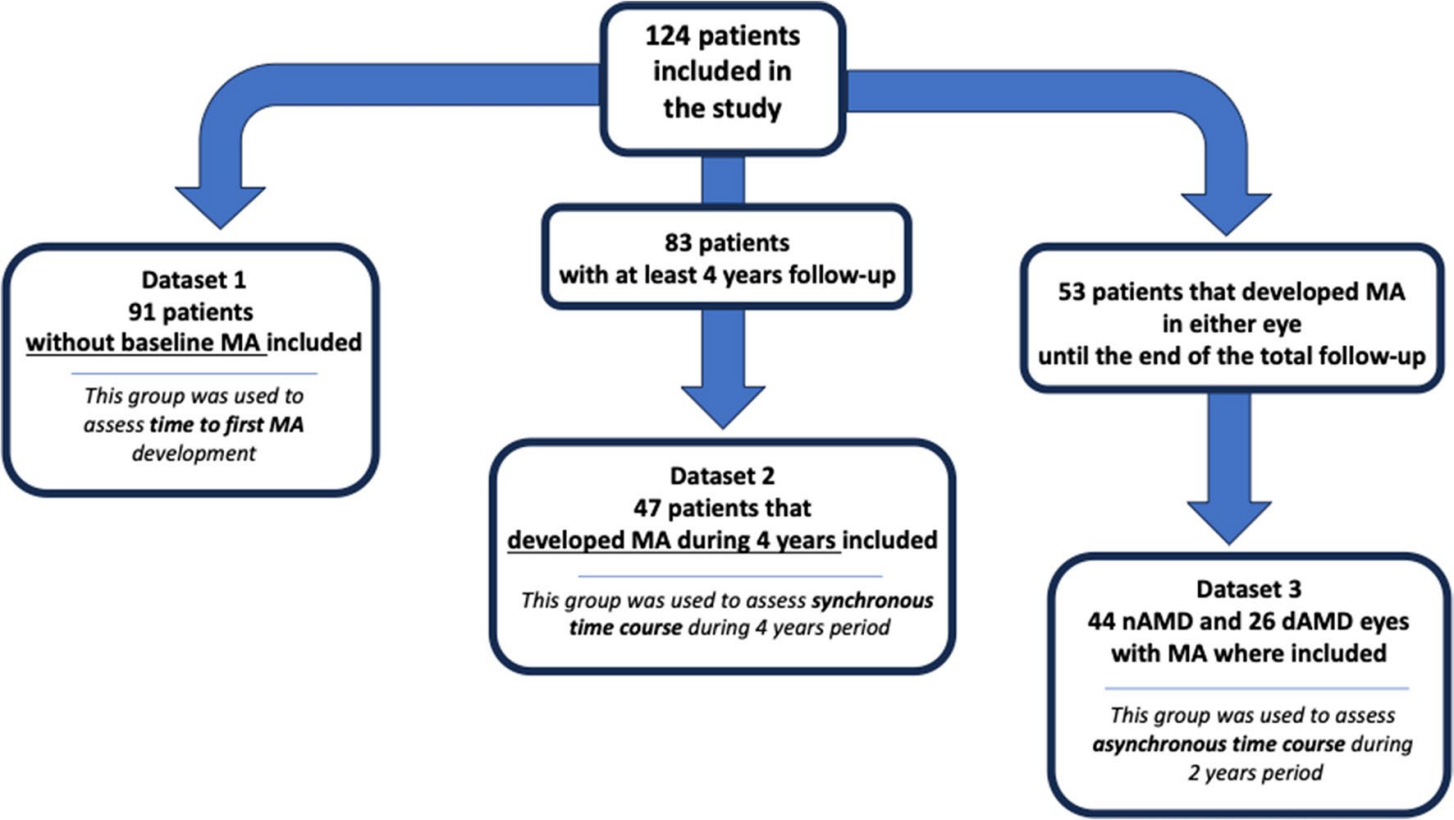
Flowchart, showing the three separate datasets used in the study. Macular atrophy (MA)

For analysis of this study, data were divided into three datasets as shown in Fig. 2.

The first dataset included patients without baseline MA in both eyes; in this group of patients, data were transformed for cross-tabulation, and we compared the time of MA first detection in nAMD eye and dAMD eye. The second dataset included patients with a follow-up duration of over 4 years. In this group of patients, we estimated the synchronous time course of MA in nAMD eyes and dAMD eyes, which also allowed us to detect possible time-eye interactions. Finally, in the third dataset, we included patients that developed MA until the end of their follow-up in either eye; we used this dataset to estimate the absolute values of MA growth rate and compare MA in nAMD and dAMD eyes in an asynchronous manner.

### Macular atrophy progression rate

The progression rate of MA was assessed in the synchronous and asynchronous datasets (datasets 2 and 3 respectively).

#### Synchronous MA analysis

Initially, we estimated synchronous MA time course in patients who developed MA within the 4-year follow-up in nAMD eye and we compared it to the progression rate of any MA in their fellow dAMD eye during the same time period. The NIR and SD-OCT images of both eyes in all patients with MA in the last visit of their nAMD eye were examined for NIR and OCT criteria for atrophy starting from baseline visit and the area of MA was measured. The progression rate of MA (mm^2^/year) was calculated as the difference of MA area in final visit minus MA area in baseline visit divided by the number of years elapsed from baseline visit to final visit. A square root transformation (SQRT, mm/year) was performed to address skewed data as follows: square root of MA area at final visit minus square root of MA area at baseline visit; then, the difference divided by the number of years elapsed from baseline visit to final visit. This approach offered the advantage of direct paired comparison of synchronous MA time course in the two eyes of each of these patients during the specific period of his/her follow-up.

#### Asynchronous MA analysis

Then, we estimated the asynchronous MA growth rate in all patients that developed MA by the last visit of their total follow-up in either eye; we used as timepoint zero the first incidence of atrophy, which might not be the same in the two eyes. All NIR and SD-OCT images were examined to find the first visit in which NIR and OCT criteria for atrophy were met (visit 0). The area of MA was measured at this visit and all subsequent visits. The growth rate of MA (mm^2^/year) was calculated as the difference of MA area in final visit minus MA area in visit 0 divided by the number of years elapsed from visit 0 to final visit. A square root transformation (SQRT, mm/year) was performed to address skewed data as described above. In this approach, a comparison is not constrained within the same time period for nAMD and dAMD eyes, as measurements start whenever MA is first detected for each eye. The growth rates of MA in nAMD and dAMD eyes were compared in an unpaired and asynchronous manner. We used patients that had completed at least 2 years of follow-up with atrophy for this analysis. Although unpaired, this approach allowed us to estimate the absolute values of MA growth rates in nAMD and dAMD eyes excluding zero atrophy from calculations in line with most similar works in the literature. Within this 3rd dataset, we also performed a subgroup analysis using a smaller number of patients who developed bilateral atrophy and permitted a within-subject analysis of asynchronous MA growth rate in their nAMD and dAMD eyes.

### Statistical analysis

Summary descriptive statistics are reported as means and standard deviations (SD) or percentages, as appropriate. Bland–Altman methods were used to assess interobserver agreement. For the analysis of MA enlargement, we used square root transformation as this reduces the association between MA enlargement and the MA at baseline, improving the linearity of the data [27]. Appropriate multivariate ANOVA models were used to identify time, eye, and interaction effects on MA. Pearson’s correlation coefficients were employed to assess the association of MA progression with the number of anti-VEGF injections. MA incidence rates were computed using Kaplan–Meier product limit estimate curves. All analyses were conducted at the two-sided 5% level of significance using IBM-SPSS for Windows 25.0 (Chicago, IL, USA).

## Results

### Characteristics of study patients

We collected 124 patients (248 eyes) that met the inclusion/exclusion criteria. Forty-three patients were from the University Hospital of Heraklion, 58 from the General Hospital of Athens Korgialenio-Benakio, and 23 from the Attikon University Hospital of Athens. The follow-up duration was ranging from 2 to 8 years with an average of 3.85 years.

Table 1 shows the demographic and clinical characteristics of our study sample; number of injections received in nAMD eye, BCVA at baseline and at final follow-up, for nAMD eye and dAMD eye respectively. The mean age of patients was 78.58 ± 8.33 years. Fifty percent of patients were female. According to FFA, 70.96% of nAMD eyes had type I CNV, 26.61% type II, and 2.41% type III. All patients followed a PRN or TREX protocol. The mean baseline BCVA for the nAMD-E was 0.56 ± 0.31 and for the fellow dAMD eyes was 0.19 ± 0.20 logMAR.

**Table 1:** Demographic and clinical characteristics of study patients

Patients (eyes)	124 (248)
Age (mean ± SD, years)	78.58 ± 8.33
Male:female	62:62
Choroidal neovascularization type in the treated eye (*n*, %)	
Type 1	88 (71)
Type 2	33 (26.6)
Type 3	3 (2.4)
Follow-up period (mean ± SD, years)	3.85 ± 1.77
Number of injections in the treated eye (mean ± SD)	15.6 ± 9.6
Baseline VA treated eye (mean ± SD, logMAR)	0.56 ± 0.31
Final BCVA-treated eye (logMAR)	0.57 ± 0.32
Baseline VA fellow eye (mean ± SD, logMAR)	0.19 ± 0.20
Final VA fellow eye (mean ± SD, logMAR)	0.29 ± 0.30

Patients’ data were divided into three datasets as explained in “Patients and methods.” Dataset 1 included 91 patients (182 eyes) with no baseline atrophy out of the total 124 patients. This group of patients was analyzed for the first detection of macular atrophy during time comparing nAMD-E and dAMD-E. Dataset 2 included 47 patients (94 eyes) who had completed at least 4 years of follow-up and developed MA, regardless of the presence or not of baseline atrophy. This group of patients was analyzed for the synchronous time course of macular atrophy in nAMD and dAMD eyes. Dataset 3 included 70 eyes (44 nAMD eyes and 26 dAMD eyes) that developed MA until the end of their follow-up; we used this dataset to calculate the absolute values of MA growth rate and compare wAMD with dAMD eyes in an asynchronous manner. Figure 2 represents a flow chart with the groups of patients that were included in the study.

### Interobserver agreement

Interdepartmental raters’ agreement analysis revealed that the mean difference between raters was 0.065 mm^2^ with 95% Bland–Altman confidence limits − 0.97 to 1.1mm^2^, with all but three of the differences falling within 0.5mm^2^; so, the overall agreement between the MA measurements on NIR images by the two graders was good.

### Time effect of macular atrophy development

**Fig. 3:**
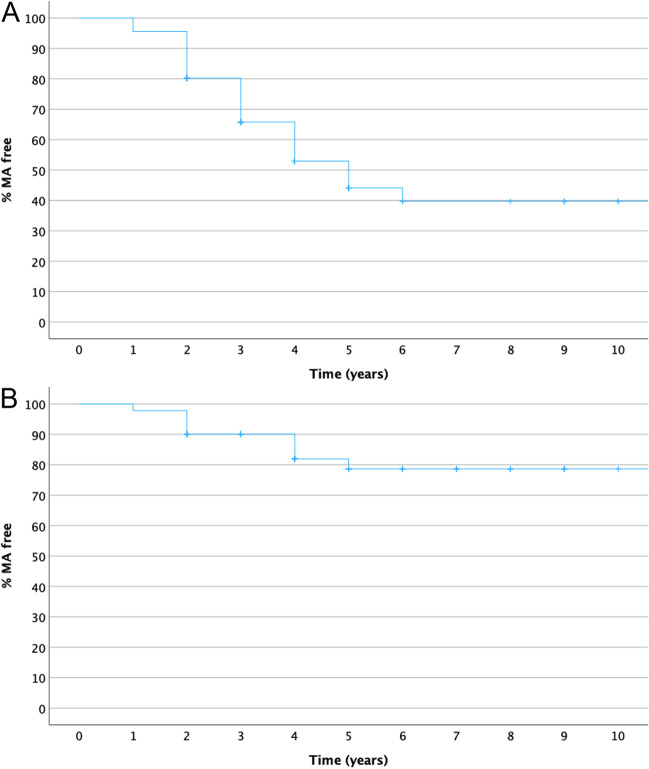
Kaplan–Meier survival curve showing the reduction by time of the percentage of neovascular age-related macular degeneration eyes (**A**) and fellow dry age-related macular degeneration eyes (**B**) without macular atrophy (MA)

The time effect of macular atrophy was evaluated in eyes with no MA at baseline in both eyes (dataset 1). Ninety-one (91) patients (91 nAMD eyes and 91 dAMD eyes) were included in this analysis. Macular atrophy first detection in nAMD eyes increased significantly from year 2 to year 6 in comparison to dAMD eyes (Fig. 3). During the follow-up period, 45.1% (41/91) of nAMD eyes developed MA, compared to 16.5% (15/91) of fellow eyes (*p* < 0.001). No significant effect of sex (*p* = 0.41) or age (*p* = 0.50) was found at the time of MA development.

### Macular atrophy synchronous time course in nAMD and dAMD eyes

The synchronous time course was evaluated in eyes that completed at least 4 years of follow-up (dataset 2). Eighty-three (83) patients met the inclusion criterion. Out of the total 83 patients, 36 did not have any atrophy at the end of their follow-up. The remaining 47 patients were used for the analysis.

**Fig. 4:**
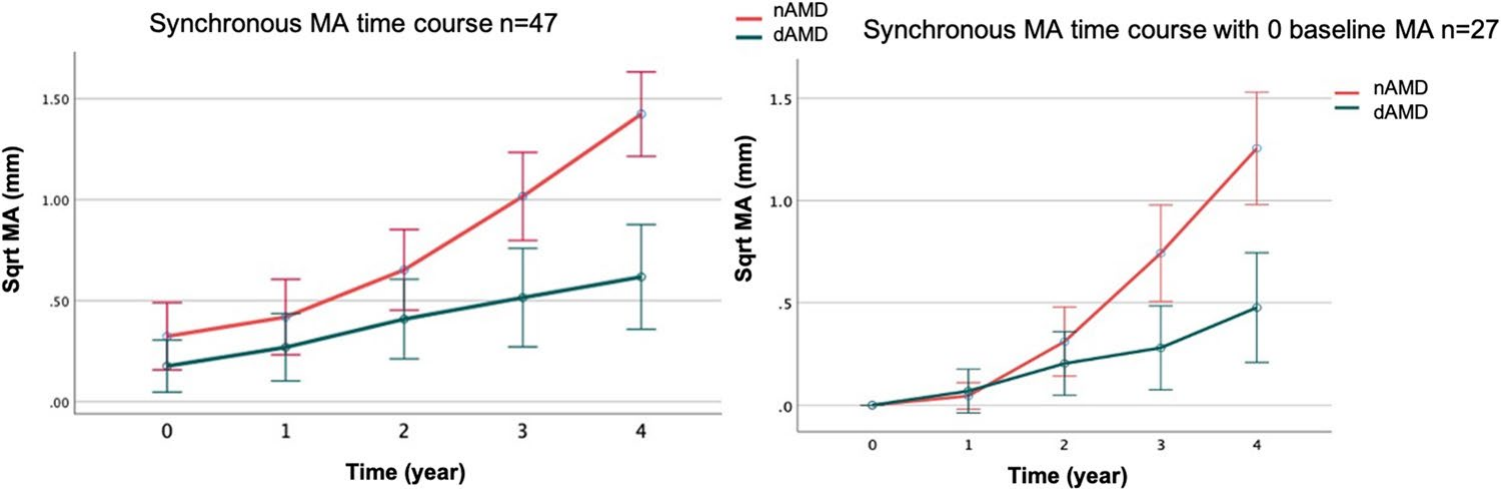
Synchronous time course macular atrophy increase in square root transformation (Sqrt MA) for neovascular age-related macular degeneration eyes and fellow dry age-related macular degeneration eyes over a time period of 4 years. Left graph represents the synchronous atrophy time course for the total 47 patients (red line 47 nAMD eyes and green line 47 dAMD eyes). Right graph represents the synchronous atrophy time course when baseline atrophy is zero (red line 27 nAMD eyes and green line 27 dAMD eyes)

MA progression rate in nAMD eyes that developed atrophy until the end of the follow-up was compared with the progression rate of any MA in their fellow dAMD eyes in a synchronous manner. All fellow eyes (even those that did not develop atrophy) were included in this analysis in a paired manner. Repeated measures ANOVA with 2 within factors [time with 5 levels (years 0 to 4 years) and eye with two levels (nAMD-E, dAMD-E)] revealed significant time (*p* < 0.001), eye (*p* = 0.003), and time-eye interaction (*p* < 0.001) effects. More specifically, the time effect was more pronounced in nAMD eyes than in dAMD eyes; in other words, MA progressed faster in nAMD-E than in dAMD-E after the 1st year of follow-up, causing gradually increasing significant differences between the two groups from year 2 onward. Indeed, the average synchronous progression rate in nAMD eyes was 0.275 mm/year for the time period of follow-up, versus 0.110 mm/year in their fellow dAMD eyes (*p* < 0.001) during the same period. More specifically, annual change is estimated as follows: 0.10 mm for year 1, 0.23 mm for year 2, 0.37 mm for year 3, and 0.40 mm for year 4 for nAMD eyes and 0.09 mm for year 1, 0.14 mm for year 2, 0.11 mm for year 3, and 0.10 mm for year 4 for dAMD eyes (Fig. 4).

### Baseline macular atrophy in nAMD eye; zero vs non-zero

Noteworthy differentiations emerged when we examined synchronous MA time course in those patients of dataset 2 who started with no baseline atrophy in the nAMD and dAMD eye (27 patients) versus those with non-zero baseline atrophy in the nAMD and dAMD eye (6 patients).

**Fig. 5:**
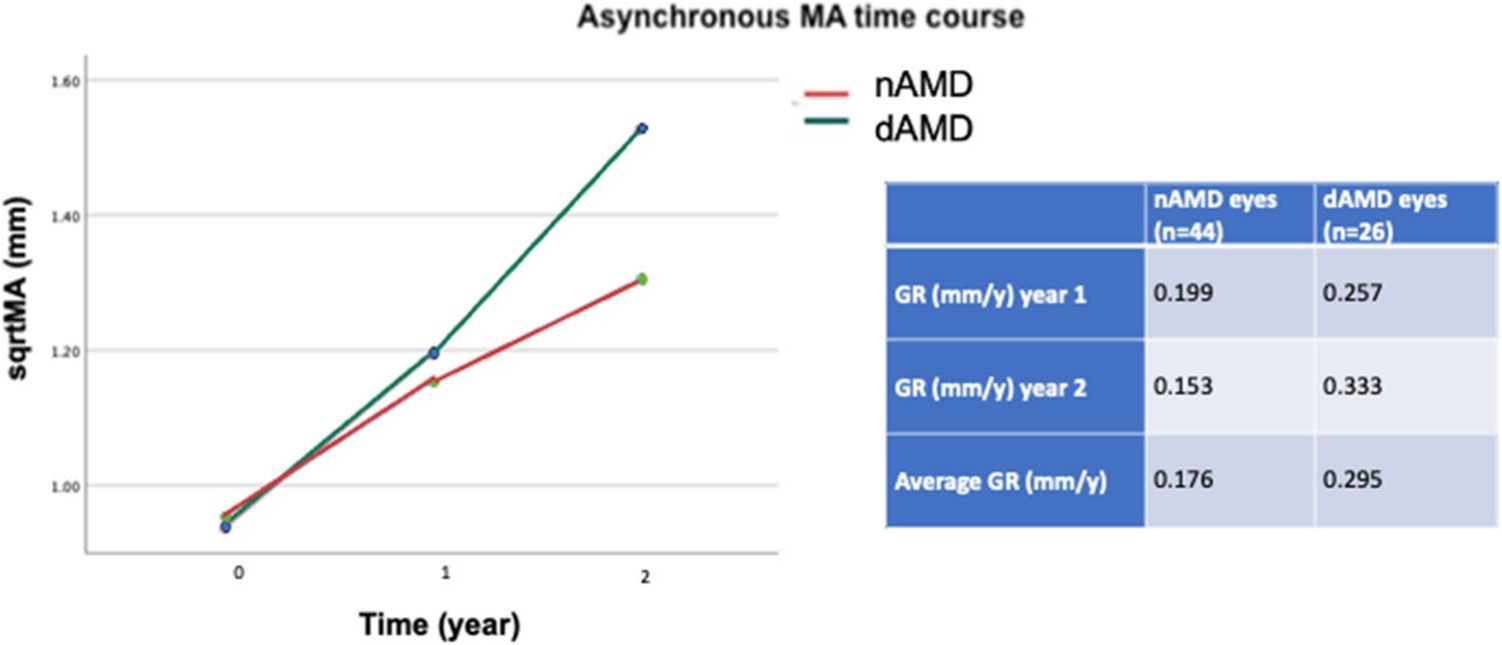
Asynchronous time course macular atrophy increase in square root transformation (sqrt MA) for neovascular age-related macular degeneration eyes (nAMD, *n* = 44, red line) and dry age-related macular degeneration eyes (dAMD, *n* = 26, green line) over a time period of 2 years. The table on the right shows MA growth rate when measured from time of first appearance; growth rate (GR)

Focusing on the subset of the 27 patients who started with no baseline MA in both eyes, we see the previously described pattern in a more pronounced manner, especially for the time-eye interaction effect. After a relatively slow growth in the nAMD-E at year 1 (0.045 mm vs 0.069 mm), MA growth in the nAMD eye is much faster than the fellow eye in subsequent years (0.310 mm vs 0.204 mm for year 2, 0.743 mm vs 0.280 mm for year 3, and 1.255 mm vs 0.477 mm for year 4). Indeed, the average synchronous growth rate in nAMD eyes was 0.314 mm/year versus 0.119 mm/year in the dAMD-E (*p* < 0.001) (Fig. 5).

On the other hand, 6 patients presented non-zero MA at baseline, in both eyes. Although this limited group of patients has insignificant statistical value, interestingly in this group, there is a trend for the eye effect, with the treated eye having more atrophy, with no interaction present; i.e., the synchronous growth in MA follows a more or less similar path for both eyes (1.317 mm vs 0.779 mm for year 2, 1.689 mm vs 2.106 mm for year 3, 1.843 mm vs 2.134 mm for year 4).

In summary, significant MA synchronous growth differences between nAMD and dAMD eyes are observed in the zero baseline MA group.

### Macular atrophy asynchronous growth rate in nAMD and dAMD eyes

We used dataset 3 to estimate the MA growth rate in nAMD eyes that developed atrophy until the end of the follow-up (*n* = 44) and MA growth rate in dAMD that developed atrophy until the end of the follow-up (*n* = 26) using as time point 0 the first time that atrophy was detected. All eyes included had at least 2 years of follow-up with atrophy. At the end of the 2nd year with atrophy, the average asynchronous growth rate in nAMD eyes was 0.176 mm/year versus 0.295 mm/year in their fellow dAMD eyes (Fig. 5). A significant time (*p* < 0.001) effect and time-eye interaction (*p* = 0.015) were found, with MA progressing significantly in both eyes but rising more rapidly in dAMD than nAMD eye, especially from end of year 1 to end of year 2.

### Paired comparison of macular atrophy asynchronous growth rate in nAMD and dAMD eyes

For this analysis, we used a subgroup of dataset 3 in which patients had developed bilateral MA. Again, a minimum of 2 years of follow-up with atrophy was required. A total of 17 patients were included. A paired within-subjects analysis was performed. At the end of the 2nd year with atrophy, the average asynchronous growth rate in dAMD eyes was higher compared to nAMD eyes (0.309 mm/year vs. 0.207 mm/year respectively). However, although the time effect was significant for both eyes (*p* < 0.001), no eye or time-eye interaction effects were found in this sub-analysis.

### Correlation of macular atrophy area with number of injections

To investigate if there is a relationship between MA areas estimated at the 4-year time point with the total number of injections of patients of dataset 2, we conducted paired *t* test. According to our results, there is no correlation between MA area and total number of injections (Pearson = 0.143, *p* = 0.196). We repeated the analysis using square root transformation and found the same results with no correlation between MA and number of injections (Pearson = 0.105, *p* = 0.344).

Furthermore, we checked whether the number of injections is correlated with the change in MA at 4 years in the non-zero group. It seems that there is a moderately strong negative significant correlation (*r* =  − 0.548) between the number of injections and change in MA at 4 years (more injections less progression to MA).

## Discussion

In this study, we evaluated atrophy development and progression in nAMD eyes treated with anti-VEGF in comparison to their fellow eyes with dAMD. Our study was designed so that we followed the behavior of the atrophic component in patients that have developed neovascular AMD in one eye, while they remain in the dry AMD spectrum in their other eye. We have selected our group on the basis of this asymmetry, and our study focused on a specific time period for each patient that this asymmetry was present. Thus, out of the total number of our patients, 45% of nAMD eyes exhibited MA until the end of the follow-up, compared to only 16.5% of their fellow eyes with dAMD during the same time period. Our findings for nAMD eyes were similar to Grunwald et al. [8], who reported a MA cumulative incidence rate of 38% during the 5 years of the CATT study in eyes with nAMD, which was much lower than the 98% MA reported by the SEVEN-UP study, following participants for up to 8 years [28]. This large difference may be attributed to the variation in definitions and assessment of atrophy, as well as in the extended follow-up time in the latter study. MA first detection in treated eyes with nAMD increased significantly from year 2 to year 5 compared with fellow eyes. This observation suggests that year 2 of the follow-up period seems to be a crucial time point for the development of MA in eyes with nAMD treated with intravitreal anti-VEGF injections, although one should consider that the specific time points could not be precise especially in retrospective studies. When we compared the time course of MA in both eyes of patients that completed a 4-year follow-up using a synchronous paired comparison, nAMD eyes presented an overall change rate of MA which was significantly faster compared to their fellow dAMD eyes. This difference was more pronounced after the 1st year of follow-up indicating that the time effect is more pronounced in nAMD eyes than in their fellow dAMD eyes. These findings demonstrate that in patients whose eyes maintain asymmetry in regard to the neovascular component for a certain time period, this asymmetry tends to be present for the atrophic component of their disease too, during this period.

Several reasons may explain the difference in MA development and progression in the nAMD and dAMD eyes of the same patient during a particular time period. Since both conditions share common pathophysiological background, one could attribute the higher incidence and higher rate of change of atrophy we found in the group of nAMD eyes, to the fact that during our follow-up period, these eyes suffered a more advanced AMD stage compared to their fellow dAMD eyes. This underlines the possible role of local factors in the development and progress of AMD, which may explain the asymmetry in presentation and temporal evolution of both the neovascular and atrophic components of the disease in the two eyes [29]. Mann and coworkers [30] have noticed that asymmetry in AMD presentation tends to be more pronounced in the late stages of the disease and hypothesized that the later manifestations of AMD may be affected by as yet unknown stochastic factors affecting the eyes separately, acting at different times during the patient’s life.

Prolonged VEGF inhibition is one of the local factors that have been implicated in MA development in nAMD patients under anti-VEGF therapy [7, 30, 31]. In our study, there was no correlation between MA area and total number of injections in eyes with nAMD. Our results are close to IVAN and HARBOR studies that did not show an association between injection frequency and new-onset or worsening of MA over 24 months [32]. Accordingly, Sadda et al. [13] reported that monthly treatment was not found to be a risk factor for MA development over 24 months. Furthermore, Christakis et al. [33] evaluated the natural history of MA in untreated nAMD eyes of AREDS and found a relatively high incidence of MA, increasing linearly over time and affecting half of the eyes by 8 years. Based on their findings, these authors suggested that factors other than anti-VEGF therapy are involved in atrophy development, including natural progression to GA [33]. Other local factors such as the absence of SRF, the presence of intraretinal cysts, reticular pseudodrusen, type III CNV, nascent GA, increased central foveal thickness, and drusen volume, as well as the collapse of pigment epithelium detachment, have been associated with GA development in eyes with nAMD [10–14].

In our study, the absolute value of growth rate in nAMD eyes (starting from the first atrophy incidence) was found to be 0.176 mm/year for a 2-year follow-up. In the HARBOR study, the growth rate in nAMD eyes was found to be 0.39 mm/year [34], while Grunwald et al. [8] reported a progression of MA at 0.30 mm/year, which is higher than our findings. This large difference may be attributed to the variation in definitions and assessments of atrophy. Of note, in the nAMD eyes with MA at baseline, the MA growth rate was relatively stable during a 4-year period of observation, compared to those which did not have MA at baseline, where the MA synchronous growth rate was found to be much faster between year 1 and 4 of the follow-up period. This finding is consistent with previous studies in the literature, declaring that the MA progression rate decreases with increasing baseline MA [35, 36]. As for the absolute value of growth rate in dAMD eyes (starting from the first atrophy incidence), we found it to be 0.257 mm/year over a 2-year follow-up period. Differences are observed in the literature in eyes with dry AMD when compared with the dAMD eyes of our study. Proxima A reported a growth rate of 0.35 mm/year [18] over a period of 24 months, while Kim et al. [37] 0.27 mm/year, and Keenan et al. [38] 0.27 mm/year. This could be explained by differences in study design, as well as differences in number of patients and differences in imaging techniques used to grade MA.

Thus, when MA growth rate of our patients was measured from the first atrophy incidence in an asynchronous manner, it was found to be higher in dAMD eyes compared to nAMD eyes. Atrophy progressed significantly both in nAMD and dAMD eyes but raised more rapidly in dAMD eyes, especially from end of year 1 to end of year 2. Even when we used a paired asynchronous comparison in a smaller group of 17 patients that developed bilateral atrophy (both in their nAMD and the dAMD eye), we found again a higher growth rate in dAMD eyes compared to their fellow nAMD eyes, although this time the difference did not reach statistical significance. Our findings agree with the findings of Airaldi et al. [20] who compared enlargement rates of macular atrophy in eyes with nAMD vs. eyes with dAMD for a period of 5 years in two different groups of patients. They also found that the presence of a neovascular component was associated with a slower progression of MA over time [20]. The main difference in this work compared to ours is the use of two different groups for the study of nAMD and dAMD growth rates. We believe that the within-subjects methodology of our work makes this finding stronger. Finally, in their work about the natural history of geographic atrophy (GA) secondary to age-related macular degeneration, Holekamp et al. [18] analyzed the results from the Proxima A and B clinical trials and included a subgroup analysis where data of GA progression rate in fellow eyes with nAMD were presented. Although they did not provide details in methodology and they did not specifically compare study eyes to their fellow nAMD eyes, the authors present a graph showing that during the 24 months of follow-up, the mean change of GA surface in the subgroup of nAMD eyes was very similar to study eyes. It is important to remember that Proxima trials were designed to study GA and their study eyes were required to have advanced disease with a minimum surface of GA [18], and this is not in analogy to our study where most of the fellow dAMD eyes had intermediate AMD.

All these findings along with ours indicate that while patients with nAMD in one eye and dAMD in their fellow eye, whose eyes are followed for a specific time period in a synchronous manner, tend to show more atrophy in their treated nAMD eye, when MA does appear in their dAMD eye, it grows faster than in the treated nAMD eye. This finding may support the hypothesis of a possible protective role of neovascularization over MA growth [20]. Also, in combination with our findings of the absence of correlation between the number of injections and MA area, it provides additional indication against the role of anti-VEGF agents as accelerators of MA.

As far as we can say, our work is one of the very few that a methodology of within-subjects comparison of MA progression in nADM and fellow dAMD eyes is used. We believe that this approach offers a unique perspective by eliminating factors (genetic, environmental, etc.), which may interfere with the pathophysiology of such a complex disease as AMD [18, 21]. Searching the literature, we were able to locate only one work that was presented at the 2020 ARVO annual meeting in which Grobben et al. [21] adopted a similar “within-subjects’ comparison” between treated nAMD eyes and fellow non-nAMD eyes. They found that the risk of developing new MA in nAMD eyes during 5 years of therapy was almost twice greater than that of fellow dry AMD eyes, and this is in agreement with our findings [21]. It is important, however, to notice that the authors of this work based their MA progress measurements on color photos and fluorescein angiograms. Moreover, making a direct comparison with our methodology was not possible since our only reference was the ARVO abstract of this work.

Potential limitations of our study are related to its retrospective nature. In a prospective design, MA measurements could be assessed with more accuracy using a standardized OCT scanning protocol with dense scans of the entire atrophic area. In this study, SD-OCT B-scans that examined to confirm MA on NIR images were representative of the atrophic areas. However, it has been reported that with the use of the combination of the two modalities (NIR + SD-OCT images), MA can be assessed with more accuracy in measuring atrophic areas [23, 39]. In our study, this was justified by the high interdepartmental raters’ agreement. Denser and consistent scanning protocols could be implemented in future studies permitting the evaluation of the entire atrophic area against CAM criteria. Another limitation pertains to the fact that nAMD cases already treated with anti-VEGF and with co-existent MA at baseline were included in the study. The optimal design would focus on treatment-naïve patients with nAMD without MA at baseline. Finally, this study was designed to compare macular atrophy in eyes treated for wet AMD, with their fellow dry AMD eyes in a specific time period. Since AMD can present temporal asymmetry between the two eyes, some of our findings could differ if the follow-up was longer, and this should be addressed in future studies. All-in-all, the comparison of our findings regarding the incidence of macular atrophy in treated and fellow eyes with other studies should be interpreted considering all methodological differences of the works.

## Conclusion

In conclusion, in this study, we provide important information on MA progression in AMD eyes comparing treated nAMD with fellow dry AMD eyes in a real-life data setting. When followed in a synchronous manner, treated nAMD eyes tend to show more atrophy compared to their fellow dAMD eyes. This indicates that in patients that, for a certain time period, maintain asymmetry between their two eyes in regard to neovascularization presence, this asymmetry tends to exist for the atrophic component of their disease too, during this period. However, when macular atrophy does appear in the dAMD eyes, it has a significantly higher growth rate compared to nAMD eyes. Future works could focus on the one hand in elucidating local or other factors that underlay the asymmetry between the two eyes, and on the other, in investigating the possible protective role of neovascularization over MA growth. Although our findings indicate the absence of a correlation between the number of injections and MA in treated eyes, this study was not specifically designed to answer this question, and as a result, more research may be necessary to clarify this matter.
